# Multifaceted roles and regulatory mechanisms of MYB transcription factors in plant development, secondary metabolism, and stress adaptation: current insights and future prospects

**DOI:** 10.1080/21645698.2025.2559489

**Published:** 2025-09-15

**Authors:** Muhammad Imran, Qiufei Wu, Chen Guanming, Lixia Zhou

**Affiliations:** a State Key Laboratory of Tropical Crop Breeding, Chinese Academy of Tropical Agricultural Sciences, Haikou, China; b Coconut Research Institute, Chinese Academy of Tropical Agricultural Science, Wenchang, China; c School of Breeding and Multiplication (Sanya Institute of Breeding and Multiplication), School of Tropical Agriculture and Forestry, Hainan University, Sanya, China

**Keywords:** DNA binding domain, MYB gene, plant stress, transcriptional regulation

## Abstract

MYB transcription factor family represents one of the largest and most functionally diverse groups of regulatory proteins in plants, playing a crucial role in controlling genes involved in growth, development, and stress responses. MYB proteins are characterized by a conserved N-terminal DNA-binding domain. They are classified based on the number of R repeats, and possess a variable C-terminal region that determines their specific functions. In response to environmental signals, MYB proteins bind to specific DNA elements in target promoters, acting alone or with other regulators to modulate stress-responsive pathways. These factors integrate signaling cascades involving abscisic acid (ABA), jasmonic acid (JA), brassinosteroids (BR), and reactive oxygen species (ROS), aiding plant adaptation to adverse conditions. This review explores structural features, classification, and regulatory mechanisms, focusing on their roles in salinity, drought, extreme temperatures, nutrient deficiencies, heavy metal toxicity, and pathogen defense. Additionally, we highlight the advances and potential of MYB genes as targets for engineering stress-resilient crops through breeding and genetic modification.

## 1. Introduction

Plants are continuously confronted by a complex array of environmental factors, and simultaneous exposure to abiotic and biotic stresses significantly constrains their growth, productivity, and nutritional quality.^1,2^ These combined stress factors pose a major threat to global food security,^3^ underscoring the urgency to deepen our understanding of how plants perceive and respond to multiple stresses occurring concurrently throughout their life cycle.^4,5^ Recent progress in molecular biology has provided new insights into the extremely intricate mechanisms that govern the crosstalk between abiotic and biotic stress responses, revealing how plants integrate diverse signals to mount coordinated defense strategies.^6,7^ To survive under these adverse conditions, plants have evolved sophisticated signaling networks that rapidly detect external stimuli and initiate adaptive responses.^8,9^ Central to these networks are transcription factors (TFs), which act as master regulators, orchestrating the activity of genes involved in stress responses. Emerging evidence demonstrates that TFs play a key role in mediating the interplay between abiotic and biotic stress pathways, allowing plants to fine-tune their responses to complex environmental challenges.^9,10^ TFs are regulated at multiple levels, including transcriptional and post-transcriptional levels, in response to fluctuating environmental conditions.^11–13^ These regulatory mechanisms enable transcription factors to precisely modulate target gene expression, facilitating plant adaptation to multiple concurrent stresses.^10,14^ Since TFs control many genes involved in both stress responses and development,^14–16^ they are key players in the networks that regulate plant stress adaptation.^17^ Furthermore, hormonal signaling pathways, such as those involving abscisic acid, jasmonic acid, and brassinosteroids, closely interact with TF-mediated networks, further enhancing the plant’s ability to integrate environmental and endogenous cues.^18,19^ These findings suggest that TFs not only integrate environmental signals but also help coordinate hormonal responses, enabling plants to mount effective defense and adaptation strategies under challenging conditions.^9,20^

**Figure 1.**
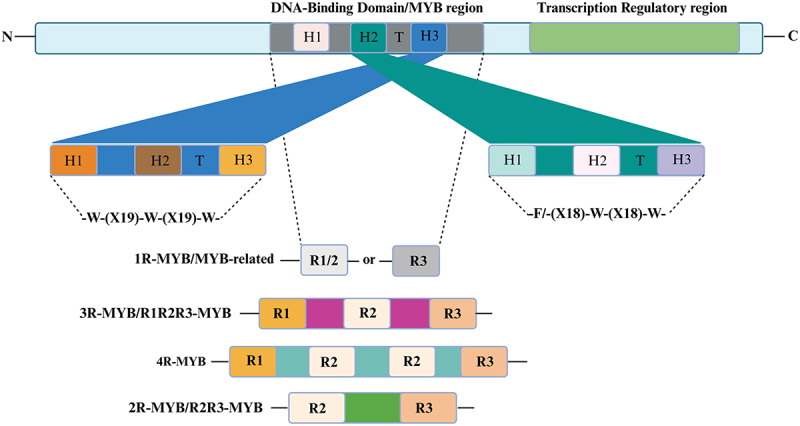
The figure illustrates the structural organization of MYB transcription factors (TFs), emphasizing the conserved DNA-binding domain and the transcriptional regulatory region. The DNA-binding domain, located at the N-terminal, typically consists of one to four imperfect repeat motifs (R1, R2, R3, or R4), each comprising three *α*-helices (H1, H2, H3), with “T” representing the *β*-turn. These repeats are characterized by conserved tryptophan residues and follow consensus sequences such as W-(X19)-W-(X19)-W or F-(X18)-W-(X18)-W. W denotes tryptophan, F phenylalanine, I isoleucine, and X any amino acid. Based on the number and arrangement of repeats, MYB TFs are categorized into four major subgroups: 1R-MYB (MYB-related), 2R-MYB (R2R3-MYB), 3R-MYB (R1R2R3-MYB), and 4R-MYB. The 1R-MYB subgroup contains a single repeat (either R1/2 or R3), 2R-MYBs contain R2 and R3 repeats and are the most prevalent in plants, 3R-MYBs have all three repeats (R1, R2, R3), and 4R-MYBs consist of four R-like domains. These structural variations contribute to the functional diversity of MYB TFs in regulating plant development, secondary metabolism, and responses to environmental stresses.

Various TF families such as WRKY, MYB, NAC, bZIP, AP2/ERF, CBF, and bHLH are prominently recognized for their roles in controlling vital activities such as cell morphogenesis, stress signal transduction, and hormone-mediated responses.^21–27^ Among these families, the MYB TFs represent one of the major groups. They are characterized by conserved structural features including the DNA-binding domain (DBD), transcriptional regulatory domain (TRD), oligomerization domain (OD), and nuclear localization signal (NLS).^28,29^ The N-terminal MYB DBD is formed by one to four imperfectly conserved repeats, each covering 50–52 amino acids, which fold into a characteristic three α-helix structure.^30,31^ The second and third α-helices form a helix-turn-helix (HTH) motif, which is stabilized by a hydrophobic core containing three regularly spaced residues, typically tryptophan. The third α-helix, referred to as the recognition helix, engages directly with the major groove of the DNA molecule.^32^ This modular structure allows MYB TFs to precisely control the expression of genes essential for plant growth, development, and adaptation to stress.^33^ Based on homology to animal c-MYB’s R1-R3 repeats, plant MYBs are classified into four groups: 1 R-MYB/MYB-related, 2 R-MYB/R2R3-MYB, 3 R-MYB/R1R2R3-MYB, and 4 R-MYB, with R2R3-MYB being the most abundant and functionally diverse subclass across species (Fig. 1).^34,35^ The functional breadth of MYBs was first highlighted by the cloning of *ZmMYBC1* in maize,^36^ followed by discoveries in *Arabidopsis thaliana*,^37^ rice (*Oryza sativa*),^38^ soybean (*Glycine max*),^39^ and sugar beet (*Beta vulgaris*),^40^ revealing dramatic lineage-specific expansions from 69 members in *Prunus avium*^29^ to 680 in *Brassica napus*.^41^ Despite their numerical dominance, R2R3-MYBs R2R3-MYB proteins, present in both monocotyledonous and dicotyledonous plants, exhibit conserved roles in phenylpropanoid biosynthesis, glucosinolate metabolism, and stress adaptation, underscoring their evolutionary significance (Table 1).^33,60,61^ Several previous reviews have addressed the roles of MYB transcription factors, primarily focusing on their involvement in plant development (e.g., cell differentiation, organ formation) and metabolic pathways such as flavonoid and phenylpropanoid biosynthesis.^62,63^ Other works have summarized MYB functions related to specific stress responses, including drought and pathogen resistance, often emphasizing individual species or pathways.^64,65^ However, existing reviews tend to treat these functions in isolation, lacking a comprehensive integration of MYB structural diversity with their multi-layered regulatory roles across diverse stress conditions and species. In contrast, this review innovatively synthesizes recent advances in MYB structural features, their contributions to complex multi-stress regulatory networks, and cross-species conservation patterns. By bridging developmental, metabolic, and stress-related functions, we provide a holistic perspective that highlights MYBs as central coordinators of plant adaptation mechanisms. This integrative approach not only advances fundamental understanding but also provides a strategic roadmap for future research and crop engineering, particularly in developing stress-resilient crops to address agricultural challenges in China.

**Table 1.** Distribution and characterization of MYB transcription factor (TF) genes across various plant species.

Species	Gene name	Number of total genes	Reference
*Phaeodactylum tricornutum*	*PtMYB*	26	^42^
*Gossypium hirsutum*	*GhMYB*	646	^43^
*Acer rubrum*	*ArMYB*	393	^44^
*Solanum tuberosum* L.	*StMYB*	158	^45^
*Cocos nucifera*	*CnMYB*	214	^46^
*Elaeis guineensis*	*EgMYB*	159	^47^
*Ipomoea batatas*	*IbMYB*	296	^48^
*Ipomoea trifida*	*ItMYB*	430	
*Ipomoea triloba*	*ItMYB*	411	
*Ipomoea nil*	*InMYB*	291	
*Ipomoea purpurea*	*IpMYB*	226	
*Ipomoea cairica*	*IcMYB*	281	
*Ipomoea aquatic*	*IaMYB*	277	
*Cucurbita moschata*	*CmoMYB*	175	^49^
*Curcuma wenyujin*	*CwMYB*	88	^50^
*Sorghum bicolor*	*SbMYB*	210	^51^
*Liriodendron chinense*	*LchiMYB*	190	^52^
*Melaleuca alternifolia*	*MaMYB*	219	^53^
*Capsicum annuum*	*CaMYB*	235	^54^
*Panax notoginseng*	*PnMYB*	123	^55^
*Pennisetum glaucum*	*PgMYB*	1133	^56^
*Chrysanthemum seticuspe*	*CsMYB*	162	^57^
*Chrysanthemum lavandulifolium*	*ClMYB*	220	
*Chrysanthemum ×morifolium*	*CmMYB*	722	
*Helianthus annuus*	*HaMYB*	386	
*Lactuca sativa*	*LsMYB*	280	
*Ziziphus mauritiana*	*ZmMYB*	56	^58^
*Ziziphus jujuba*	*ZjMYB*	60	
*Morella rubra*	*MrMYB*	174	^59^

## 2. Mechanisms of Transcriptional Regulation

The critical functional mechanisms of MYB TFs in plants are largely mediated by their conserved R2R3 DNA-binding domain, which facilitates precise recognition and binding to specific *cis*-regulatory sequences in the promoters of target genes.^22,66^ Upon binding to these motifs, MYB proteins recruit various co-regulatory complexes, including histone acetyltransferases and chromatin remodelers, that modify chromatin structure to facilitate or repress transcription.^66,67^ This dynamic modulation of chromatin accessibility allows MYB factors to function as potent transcriptional activators or repressors depending on developmental cues or environmental stimuli.^68,69^ A distinctive feature of plant MYB TFs is their capacity to assemble into combinatorial complexes with basic helix-loop-helix (bHLH) and WD40 proteins. The MBW complex exemplifies this, acting as a key modulator of essential processes such as flavonoid biosynthesis, secondary cell wall development, and responses to both biotic and abiotic stresses.^70,71^ Moreover, plant MYB activity is finely tuned by post-translational modifications, alternative splicing, and interaction with co-repressors like TOPLESS, enabling rapid and context-specific transcriptional reprogramming.^72,73^ By employing these diverse regulatory mechanisms, MYB transcription factors function as central integrators of internal developmental signals and external environmental stimuli, orchestrating the precise regulation of gene networks vital for plant growth, adaptation, and survival.^74,75^

### 2.1. MYB TFs and Their Interactions with Regulatory Upstream and Downstream Factors

MYB TFs serve as master regulators of gene expression, precisely modulating transcriptional programs through specific binding to cis-acting elements in target gene promoters. This molecular mechanism enables MYB TFs to orchestrate diverse biological processes critical for plant growth, development, and environmental adaptation.^22^ This direct DNA binding enables MYB TFs to function as molecular switches that either activate or repress transcriptional programs. MYB transcription factors are classified into various sub-groups based on their DNA-binding domains and conserved motifs.^76^ Certain sub-groups predominantly function as activators, while others act as repressors.^77^ For instance, many repressor MYBs contain conserved repression motifs such as the EAR motif (LxLxL or DLNxxP), which are absent in activators.^78^ Both activators and repressors often bind to similar MYB recognition elements (MREs) within promoter regions. However, their regulatory effects depend on additional factors such as co-factor recruitment and chromatin context.^33^ To determine whether an unknown MYB acts as an activator or repressor, researchers typically analyze its sequence for activation or repression motifs.^79^ They also perform phylogenetic comparisons with characterized MYBs, conduct transactivation assays using reporter genes, and study the expression changes of downstream targets upon MYB overexpression or knockout.^80^ In *Arabidopsis*, *AtMYB73* interacts with the promoter regions of actin-depolymerizing factor (ADF) genes. This interaction suppresses their transcription and thereby enhances actin filament stability to regulate cytoskeletal dynamics.^75^ In rice, *OsMYB30* activates transcription of *OsPAL6* and *OsPAL8*. This elevates phenylalanine ammonia-lyase (PAL) levels and promotes lignin and salicylic acid accumulation, enhancing resistance against the brown plant-hopper (*Nilaparvata lugens*).^81^ Similarly, overexpression of PaMYB9A1/2 from *Phalaenopsis aphrodite* in tobacco directly upregulates genes involved in cuticular wax synthesis and cell wall biosynthesis. This leads to increased wax deposition, improved leaf glossiness, and reinforced protection against water loss and pathogens.^82^ These compelling examples demonstrate the remarkable precision of MYB transcription factors in reprogramming transcriptional networks through specific *cis*-element recognition.^22^ This targeting mechanism is fundamental to plant development and environmental responsiveness.^75^

Beyond direct DNA binding, MYB TF activity is intricately regulated through interactions with upstream proteins. These proteins modulate their binding affinity and transcriptional outcomes, thereby fine-tuning plant phenotypes.^32^ In this context, “upstream proteins” refers to transcription factors or signaling components that act earlier in regulatory cascades. They influence the activity, localization, or DNA-binding capacity of MYB TFs. These upstream regulators include photoreceptors, kinases, or other TFs that modify MYB function in response to environmental or developmental cues. Under ultraviolet (UV) light exposure, Arabidopsis *AtMYB73/77* interacts with photoreceptors that inhibit its binding to auxin-responsive genes. This interaction suppresses hypocotyl elongation and lateral root formation.^83^ A hallmark of plant MYBs is the presence of an R3 repeat containing a bHLH-interaction domain. This domain facilitates the assembly of the MBW complex (MYB-bHLH-WD40).^64^ In *Arabidopsis*, *AtMYB75, AtMYB90*, and *AtMYB113* (also known as PAP1, PAP2, and PAP3) interact with bHLH proteins GL3, EGL3, and TT8, along with the WD40 protein TTG1. Together, they assemble the MBW complex, which strongly effects the expression of anthocyanin biosynthesis genes including *LDOX* and *DFR*, thereby promoting pigment accumulation.^84^ MYB TFs can interact with a variety of other TFs and regulatory proteins. These interactions are mediated by specific domains within the MYB protein: the R3 repeat contains a conserved motif essential for bHLH partner binding, while other regions such as the C-terminal domain or intrinsically disordered regions mediate interactions with non-bHLH partners. This domain specificity allows MYBs to form diverse regulatory complexes. Furthermore, MYB TFs can form homo- and hetero-multimers to enhance regulatory specificity and strength. For instance, in *Betula platyphylla*, *BplMYB46* forms heterodimers with several MYB partners (*BplMYB6, 8, 11, 12, 13*), collectively boosting their binding affinity to downstream genes. The co-expression of *BplMYB46* and *BplMYB13* leads to a significant upregulation of genes encoding antioxidant enzymes, including superoxide dismutase (SOD), peroxidase (POD), and glutathione S-transferase, which collectively boost reactive oxygen species (ROS) scavenging capacity and strengthen stress tolerance.^85^ Through these complex interactions, MYB transcription factors function as key integrators within regulatory networks, modulating gene expression by both direct DNA binding and interactions with other proteins, which enables plants to finely regulate their growth, development in hazards conditions.

### 2.2. MYB Participates in Regulating Signaling Pathways

Plant hormone signaling pathways, such as those involving abscisic acid (ABA), indole-3-acetic acid (IAA), and gibberellins (GAs), are intricately regulated by a complex network of transcription factors. These factors control gene expression in response to both developmental signals and environmental stimuli.^86–88^ Studies have demonstrated that the application of indole-3-acetic acid (IAA) to *Citrus grandis* juice sacs significantly downregulates the expression of *CgMYB58* and its downstream lignin biosynthesis genes, whereas treatment with abscisic acid (ABA) enhances their transcription.^89^ Similarly, under ABA treatment, *AchnABF2*, *AchnMYB41*, and *AchnMYB107* in kiwifruit are induced to bind to the ABA-responsive element (ABRE) and MYB recognition element (MRE) located in the promoter region of *AchnFHT*. This binding enables its transcriptional activation, thereby positively regulating lignin biosynthesis.^90^ In potatoes, the expression of 16 transcription factors, including *StMYB1*, is upregulated by IAA, GA3 and ABA. However, the expression of *StMYB6* was significantly suppressed under all three hormone treatments, emphasizing the functional diversity of MYBs in plant hormone responses.^45^ Beyond these hormones, MYB transcription factors also participate in jasmonic acid (JA) and ethylene (ETH) signaling pathways. For example, ETH and JA treatments upregulate the transcription level of *RhMYB108* in *Rosa hybrida*. The induced *RhMYB108* directly acts on the promoters of petal senescence-related genes *RhNAC053*, *RhNAC092*, and *RhSAG113*, thereby accelerating petal senescence.^91^ Similarly, in *Citrus sinensis*, *SINAT4* and *PIN5* are downstream target genes of *CsMYB77*. *CsMYB77* transcriptionally represses *SINAT4*, delaying ABA signaling transduction for fruit ripening, while it activated *PIN5*, reducing free IAA content in the fruit and modulating auxin signaling. Therefore, *CsMYB77* integrates abscisic acid (ABA) and auxin signaling pathways to modulate fruit ripening and determine fruit size.^60^

In addition to hormone signaling, MYB TFs participate in multiple other signaling cascades, such as the mitogen-activated protein kinase (MAPK) pathway, ROS signaling, and the phospholipase D/phosphatidic acid (PLD/PA) pathway, all of which play vital roles in plant under unfavorable condition.^92^ When cell membrane receptors perceive environmental signals, the MAPK cascade mediates phosphorylation reaction, transmitting the signal into the cell and regulating the expression of intracellular MYBs.^93^ These changes induce physiological and biochemical modifications in plants, thereby improving their capacity to respond and adapt to environmental variations.^68^ For example, in rice, the upstream kinase *OsMAPK10* phosphorylates and activates *OsRLM1*, which subsequently binds directly to the promoter of the downstream gene *OsCAD2*, thereby modulating the biosynthesis of the secondary cell wall.^94^ Additionally, phosphatidylcholine is catalyzed by PLD to generate PA, which acts as a second messenger. PA phosphorylates and activates the downstream MAPK cascade, subsequently activating the target gene MYB. The activated MYB protein assembles into a complex with bHLH and WD40 partners, directly interacting with the promoters of flavonoid biosynthetic genes enhancing flavonoid production, decreasing intracellular ROS concentrations, and consequently improving plant stress tolerance.^33^

## 3. MYB Transcription Factors in Abiotic and Biotic Stress Responses

Plants are continuously challenged by a diverse range of stress factors, including abiotic stresses such as salinity, drought, temperature variations, nutrient deficiencies, and heavy metal toxicity, alongside biotic stresses like pathogen attacks and herbivore damage. These adverse conditions significantly impair plant growth, productivity, and overall quality.^95,96^ Recent studies have underscored the pivotal role of MYB TFs in directing plant responses to these stresses, particularly highlighting their contribution to enhancing tolerance against external stressors, which has garnered substantial research attention (Table 2).^68^ They regulate stress-responsive genes by binding to target regions of the promoter, thereby modulating their transcriptional activity.^128^ The key mechanisms through which MYB TFs confer stress tolerance include: (i) maintaining cellular ionic balance by adjusting the expression of transporters involved in Na^+^ and K^+^ homeostasis; (ii) alleviating oxidative stress by upregulating ROS-scavenging systems, thus protecting cellular membranes, proteins, and nucleic acids from oxidative damage; (iii) preserving water balance by enhancing water uptake or minimizing water loss, which supports osmotic stability during drought and other stress conditions; (iv) promoting the accumulation of osmo-protectants to mitigate hyperosmotic stress effects; and (v) modulating hormonal signaling pathways, particularly abscisic acid (ABA), to activate downstream adaptive responses essential for stress resilience.^98,129^

**Table 2.** Role of MYB transcription factors in regulation of plant responses under abiotic stress conditions.

Species	Gene name	Gene family	Target genes and sites	Function	Stress response	Reference
*Arabidopsis thaliana*	*AtMYB25*	*R2R3-MYB*	*JAZ10, RD29a, DREB2C, SLAH1,*	Activate downstream stress response genes	Salinity, Permeability, Abscisic acid	^97^
	*AtMYB37*	*R2R3-MYB*	*RD22, COR15A, ABF2/3, PSII/I, RD29a,*	Enhance PSII activity and regulate energy dissipation ratio	Salinity, Drought, Abscisic acid	^98^
	*AtMYBS1*	*MYB-related*	*MAX1*	Negative regulation of the one-legged gold lactone pathway	High temperature (-)	^99^
	*AtMYB74*	*R2R3-MYB*	*NIG1, MYB102, ERF53, HSFA6a, MYB90, MYB47*	Regulate downstream target genes after activation of auxin precursor IAM	Permeability, High temperature	^100^
	*AtMYB12*	*R2R3-MYB*	*NCED, AAO, ZEP, ABA2, CAT, POD, SOD, P5CR, P5CS*, *LEA*	Increase the content of flavonoids in plants under stress conditions	Salinity, Drought, High temperature, Ultraviolet rays	^101^
	*AtMYB71*	*R2R3-MYB*	ABA response genes	Regulating plant ABA response	Abscisic acid	^102^
	*AtMYB94/96*	*R2R3-MYB*	*KCR1, KCS1/2/6, CER1/3, WSD1*	Promote the biosynthesis of plant epidermal wax	Drought, Ultraviolet rays, Strong light	^103^
*Arachis hypogaea*	*AhMYB30*	*MYB-related*	*KIN1, COR15a, RD29A, ABI2*	Upregulation of downstream stress-related gene expression involved in DREB/CBF and ABA signaling pathways	Low temperature, Salinity	^104^
*Brassica campestris*	*BcMYB111*	*R2R3-MYB*	*F3H, FLS1*	Enhanced flavonoid biosynthesis after CBF transcriptional activation	Low temperature	^105^
*Oryza sativa*	*OsMYBR57*	*MYB-related*	*OsbZIPs* regulated by interaction with *OsHB22*	Activation of transcription factor bZIP after interaction with HB22	Drought	^106^
	*OsMYB-R1*	*MYB-related*	*CAT, SOD, GPX, ABRE, LEA,*	Activate downstream stress-related genes	Drought, Chromium element, Salicylic acid, Abscisic acid, Jasmonic acid	^107^
	*OsFLP*	*R2R3-MYB*	*NAC1/6, DST*, peroxidase 24 precursor	Activate downstream related transcription factors	Salinity, Drought, Abscisic acid	^108^
*Gossypium hirsutum*	*GhMYB36*	*R2R3-MYB*	*PR1*	Activate downstream related genes	Drought, Verticillium wilt disease	^109^
	*GhMYB102*	*R2R3-MYB*	*NCED1, ZAT10*	Participate in regulating ABA biosynthesis and drought response gene expression	Drought	^110^
*Limonium bicolor*	*LbMYB48*	*MYB-related*	*DIS3, CPC-like, SOSs, GSTs, RLKs*	Regulating the expression of genes related to epidermal development and salt stress	Salinity	^111^
	*LbTRY*	*MYB-related*	*ZFP5, GL3, RHD6, LRL2/3, P5CS, RSL1, SOS1/2/3*	Upregulation of GL3/ZFP5 expression leads to competitive binding with the expressed product, altering the differentiation direction of transgenic plant epidermal cells, enhancing root hair development, and absorbing more Na+	Salinity (-)	^112^
*Carya cathayensis*	*CcMYB12*	*R2R3-MYB*	*C4H, CHI, F3H, ANR, ANS, DFR*	Participate in anthocyanin synthesis pathway	Salinity, Drought, Acid	^113^
*Hevea brasiliensis*	*HbMYB44*	R2R3-MYB	Homologous genes and interacting protein-encoding genes	Activate downstream stress-related genes	Salinity, Permeability, Abscisic acid, Drought, Methyl jasmonate, Gibberellin, Salicylic acid	^114^
*Ipomoea batatas*	*IbMYB308*	R2R3-MYB	*SOD, POD, APX, P5CS*	Activate downstream stress-related genes	Salinity	^115^
	*IbMYB73*	R2R3-MYB	*ABA2, ABI2, NCED3, AAO3, DREB1D, SnRK2.3, GER5, RD22, RD26,*	Activation of ABA dependent negative regulatory factors transcription for adventitious root growth and stress tolerance express	Salinity (-), Drought (-), Abscisic acid (-)	^116^
*Chenopodium glaucum*	*CgMYB1*	R2R3-MYB	*NHX1, HAK5, SOS1, P5CS2, POD1, SOD, CBF1, bHLH001, COR15, COR47,*	Enhance the physiological functions and stress-related gene expression of genetically modified plants	Salinity, Low temperature	^117^
*Pisum sativum*	*PsFLP*	R2R3-MYB	*AAO3, CDKA1, CYCA2, SnRK2.3, CYCA3, NCED3,*	Regulating stomatal formation, ABA synthesis, and signal transduction genes	Drought, Abscisic acid	^118^
*Populus alba × Populus glandulosa*	*PagMYB205*	R2R3-MYB	Genes related to *SOD, POD, CAT*, and root vitality	Negative regulation of antioxidant enzyme activity and root vitality	Salinity (-)	^119^
	*PagMYB151*	R2R3-MYB	Proline biosynthesis genes	Together with co expressed transcription factors, it alters root structure, promotes proline accumulation, and reduces MDA content	Salinity	^120^
*V. labrusca × V. riparia*	*VhMYB2*	R2R3-MYB	*NHX1, NCED3, SOS1/2/3, P5CS1, SnRK2.6*, *CAT1*	Activate downstream-related genes	Salinity, Drought	^121^
*Capsicum annuum*	*CaDIM1*	MYB-related	*OSR1, RAB18, NCED3* and stress responsive genes	Induced stress/ABA related gene expression	Drought, Abscisic acid	^122^
*Fagopyrum tataricum*	*FtMYB11*	R2R3-MYB	*ABA3, NCED3, CBF1, DREB2A, F3H, ANS, RD20, C4H, 4CL, DFR*	Regulating the expression of genes associated with the ABA signaling pathway, drought response, and flavonoid biosynthesis	Salinity (-), Drought (-), Abscisic acid (-)	^123^
*Apium graveolens*	*AgMYB5*	R2R3-MYB	*CRTISO, LCYB, ABA1/2, NCED6, AAO3, ERD1, RD22, P5CS1, RD29*	Enhanced β-carotene biosynthesis and subsequently induced ABA synthesis	Oxidative damage, Drought	^124^
*Setaria italica*	*SiMYB16*	MYB-related	*FAR1, 4CL1, CSE, PAL, CYP87A3, NCED3, F5H, COMT*	Modulating the biosynthesis of lignin, flavonoids, and suberin in plants	Salinity	^125^
*Vitis amurensis*	*VaMYB14*	R2R3-MYB	ABA signaling genes, LTPs, CORs, POD, and CAT	Participating in the activation of ABA signaling components and the CBF-COR-independent expression of LTP3	Salinity, Drought, Abscisic acid	^126^
*Solanum lycopersicum*	*SlMYB41*	R2R3-MYB	*SlHSP90.3*	Maintain the homeostasis of reactive oxygen species under heat stress	High temperature	^127^
*Elaeis guineensis Jacq.*	*EgMYB111, EgMYB157*	R2R3-MYB	*SnRK2.1, SnRK2.3, SnRK2.5*	Induced stress related gene expression	Salinity, Drought, Low temperature	^47^

“”- indicates negative regulation.

### 3.1. Regulation of Salt Stress Tolerance by MYB TFs

**Figure 2.**
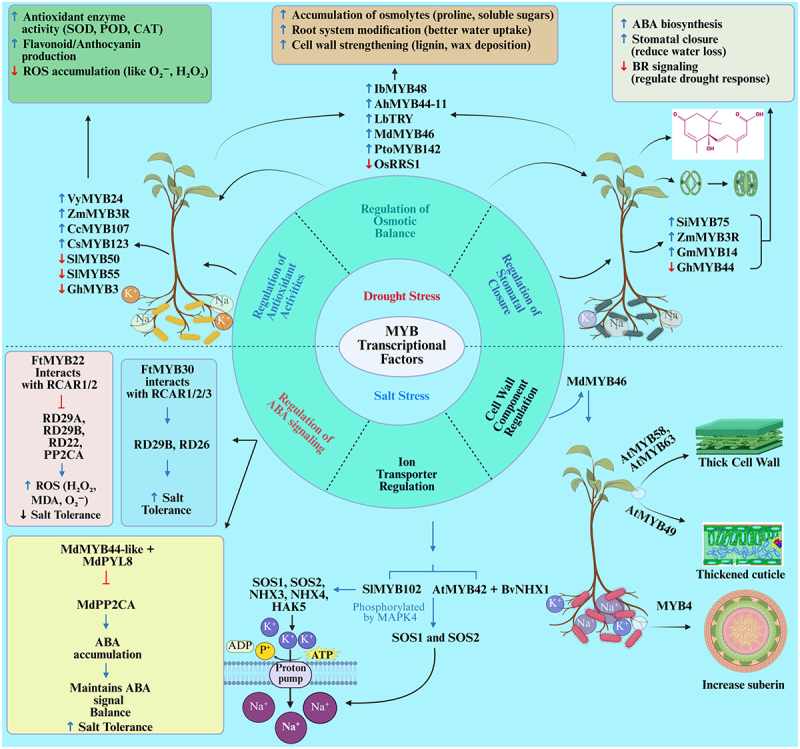
Schematic illustration of the role of MYB transcription factors in plant responses to drought and salt stress. The diagram depicts the regulatory functions of MYB transcription factors (TFs) in plant stress responses, particularly under drought and salt stress conditions. MYB TFs are shown as central regulators of various stress-related pathways, including antioxidant enzyme activities (such as SOD, POD, and CAT), ROS accumulation, and osmotic balance. Blue line arrows (↑) represent the activation or upregulation of specific processes, while downward red arrows (↓) indicate suppression or downregulation. Whereas red T-bar (–) indicates inhibition of gene function or signaling pathways.

Soil salinization has emerged as a significant global challenge to agriculture, driven by environmental degradation and improper irrigation practices, which adversely affect crop productivity.^130^ Salt stress disrupts seed germination, restricts water and nutrient uptake, and induces both ionic and osmotic stresses, collectively impairing normal plant growth and development.^131^ These stressors cause detrimental effects such as oxidative damage and ion toxicity, further compromising plant health.^132^ Extensive research has identified MYB as a key player in regulators in plant responses to salt stress, primarily through their involvement in the ABA signaling pathway.^75^ Under saline conditions, MYB proteins influence cell wall remodeling and regulate the expression of ion transporter genes, which are essential for maintaining osmotic balance and ion homeostasis. This regulatory role enables plants to adapt effectively to high salinity environments (Fig. 2).^133^

#### 3.1.1. ABA-Dependent MYB Pathways in Salinity Stress

MYB transcription factors modulate plant salt tolerance primarily via ABA-dependent signaling pathways.^22^ In *Fagopyrum tataricum*, *FtMYB22* interacts with ABA receptors *RCAR1*/*2*, destabilizing the RCAR-ABA-PP2C complex under salt stress. This interaction promotes PP2C binding to *SnRK2.3*, inhibiting its kinase activity and subsequent phosphorylation of downstream targets. It has been shown that *FtMYB22* directly binds to promoter regions containing ABRE *cis*-elements, resulting in the downregulation of stress-responsive genes such as *AtRD29A, AtRD29B, AtPP2CA*, and *AtRD22*. This repression correlates with elevated levels of MDA, hydrogen peroxide (H₂O₂), and superoxide anions (O₂^−^) in transgenic plants, along with a delayed stomatal closure response. These findings suggest that *FtMYB22* negatively regulates salt tolerance via an ABA-dependent signaling mechanism.^134^ Conversely, *FtMYB30* has been reported to directly associate with ABA receptor proteins *AtRCAR1, AtRCAR2*, and *AtRCAR3*, thereby promoting the expression of ABA-responsive genes including *RD29B* and *RD26*. This interaction decreases the sensitivity of transgenic plants to salt and ABA, ultimately enhancing their tolerance to salt stress.^135^ MYB proteins can regulate ABA signaling homeostasis under salt stress by interacting with components of the ABA pathway. For example, *MdMYB44*-like, which is highly expressed in apple leaf guard cells, binds directly to the MBS element in the promoter of *MdPP2CA*, repressing its expression. This repression is strengthened through its association with the ABA receptor MdPYL8, leading to rapid accumulation of abscisic acid (ABA) during salt stress. However, increased ABA levels trigger *MdPP2CA* to interfere with the *MdMYB44*-like–*MdPYL8* interaction, with all three components working together to maintain ABA signaling balance and thereby enhance plant tolerance to salt stress.^136^

#### 3.1.2. MYB-Controlled Cell Wall Remodeling Under Salt Stress

MYBs are essential regulators of plant salt tolerance, in part by modulating the deposition and reinforcement of cell wall constituents, including lignin and cuticular layers.^137^ Through the regulation of cell wall composition, MYB transcription factors contribute to mitigating cell turgor disruption caused by salt-induced water deficit, thereby improving plant tolerance to salinity.^138^ For instance, in Arabidopsis plants overexpressing *MdMYB46*, this transcription factor directly binds to SMRE and M46RE motifs within the promoters of lignin biosynthetic genes, as well as to the promoter regions of key transcriptional activators including *AtMYB58* and *AtMYB63*. It modulates lignin biosynthesis, increased cell wall mechanical strength, and facilitated the preservation of intracellular osmotic homeostasis, collectively enhancing salt tolerance in transgenic plants.^75,139^ Similarly, *AtMYB49* acts as an activator of genes such as *ASFT, MYB41, FACT*, and *CYP86B1*. Overexpression of *AtMYB49* in transgenic *Arabidopsis* led to the development of thicker leaf cuticles, which effectively alleviated osmotic stress induced by salinity.^140^ Furthermore, the MYB transcription factors M*YB41, MYB53, MYB92*, and MYB93 in Arabidopsis act as positive regulators of suberin biosynthesis in the cell wall. Concurrent mutations in these MYBs resulted in a marked decrease in suberin accumulation within the root endodermis, accompanied by increased water loss relative to wild-type plants, ultimately reducing salt tolerance.^141^ Conversely, certain MYB TFs act as negative regulators of lignin deposition during salt stress. For example, *AtMYB3* possesses a C-terminal LNL(E/D)L repressor motif that directly binds to the promoter sites of *C4H, PAL2, 4CL3*, and *COMT*, suppressing their expression. This repression results in decreased lignin accumulation under saline conditions, heightened plant sensitivity to salt, and a consequent reduction in salt tolerance.^142^

#### 3.1.3. Ion Homeostasis Regulation by MYBs in Saline Conditions

Salt stress leads to the excessive accumulation of Na^+^ in both roots and aerial tissues, eventually reaching toxic levels that disrupt the balance of intracellular ions.^143^ Research has shown that MYBs regulate the expression of ion transport-related genes, facilitating the compartmentalization of excess Na^+^ into vacuoles or its extrusion out of the cell, thereby enhancing the cytoplasmic K^+^/Na^+^ ratio.^144^ This response helps alleviate ionic toxicity caused by elevated Na^+^ levels, maintain cellular ion and redox homeostasis, and contribute to improved salt tolerance in the transgenic plants.^145^ For example, Studies in *Beta vulgaris* revealed that mutation of the MYB binding site in the *BvNHX1* promoter abolished its transcriptional activity^146^ This indicated that MYB transcription factors participate in activating *BvNHX1* expression under salt stress, accelerating Na^+^ compartmentalization into vacuoles and regulating sugar beet salt tolerance.^147^ Over-expression of *SlMYB102* in *Solanum lycopersicum* significantly enhanced the transcription levels of genes such as *SlSOS1*, *SlSOS2*, *SlNHX3*, *SlNHX4*, and *SlHAK5*. By expelling excess Na^+^ from the cytoplasm, compartmentalizing it into vacuoles, and enhancing K^+^ uptake, transgenic tomato leaves and roots showed reduced Na^+^ levels and increased K^+^ content under salt stress. This contributes to maintaining the Na^+^/K^+^ balance in plants, thereby improving salt tolerance in transgenic lines.^148^ Likewise, during salt stress in *Arabidopsis*, *AtMYB42* is phosphorylated and activated by *MAPK4*, enabling it to bind the promoter of *SOS2* and enhance its transcription, which in turn leads to the activation of downstream *SOS1*. This process promotes the efflux of surplus Na^+^ ions from cells, thereby supporting ion homeostasis and enhancing the plant’s capacity to tolerate salt stress.^149^

### 3.2. MYB-Mediated Responses to Drought Stress

Drought is a major environmental stress that greatly affects plant growth, development, and overall yield.^150^ Prolonged water deficit leads to the peroxidation of proteins, lipids, and nucleic acids within plant cells, rendering them inactive.^151^ Additionally, water loss causes osmotic imbalance in plant cells, severely disrupting physiological processes and normal metabolic functions.^152,153^ Research has shown that MYBs regulate plant responses to drought stress through multiple mechanisms, including enhancing ROS scavenging capacity,^75^ maintaining osmotic balance by accumulating small-molecule osmolytes, modifying root systems to improve water uptake,^154^ thickening leaf cuticles to reduce water loss,^22^ and participating in ABA-mediated regulation of stomatal movement in plant leaves (Fig. 2).^22^

#### 3.2.1. MYB-Driven Antioxidant Defense Mechanisms Under Drought Stress

Abundant studies have highlighted the involvement of MYB TFs in strengthening plant antioxidant defense systems, which include enzymatic antioxidants such as superoxide dismutase (SOD), peroxidase (POD), polyphenol oxidase, and ascorbate peroxidase, as well as non-enzymatic antioxidants including *β*-carotene, glutathione, and carotenoids.^155–157^ These antioxidants help mitigate oxidative stress induced by water deficiency, thereby enhancing the plant’s drought tolerance (Fig. 2). For instance, the overexpression of *VyMYB24* from *Vitis yanshanesis* in tobacco and *ZmMYB3R* from maize in *Arabidopsis* resulted in a marked increase in the expression of antioxidant enzyme genes such as SOD, POD, and catalase (CAT). This facilitated the efficient removal of O₂^−^ and hydrogen peroxide (H₂O₂) produced during osmotic stress, thereby improving drought tolerance in transgenic plants.^158–160^ conducted a comprehensive identification and characterization of the *CcMYB* gene family in *Cajanus cajan*, revealing that *CcMYB107* expression is significantly induced by drought stress. Functional analysis demonstrated that OE-*CcMYB107* enhances antioxidant enzyme activities, reduces peroxide accumulation, and significantly improves drought tolerance in transgenic plants compared to wild-type controls. Flavonoids, as important non-enzymatic antioxidants, effectively reduce ROS accumulation induced by drought stress.^161^ For example, *CsMYB123* from *Chaenomeles speciosa* interacted with *CsbHLH111* to activate the transcription of the anthocyanin biosynthesis gene *CHI*. Under drought conditions, *CsMYB123* over-expression lines accumulated more anthocyanins than control, effectively mitigating ROS-induced damage, protecting membrane integrity, and enhancing drought tolerance.^162^ Conversely, RNA interference-mediated silencing of *SlMYB50*, *SlMYB55* in tomato, and *GhMYB3* in cotton enhances the transcriptional activation of *4CL*, *CHS*, *ANS*, and *ANR*, leading to increased anthocyanin and proanthocyanin content, elevated antioxidant enzyme activity, and timely clearance of excess ROS in leaves and roots. This reduced oxidative toxicity to proteins, lipids, and nucleic acids, positively contributing to improved drought resistance.^163–165^

#### 3.2.2. MYB Role in Osmotic Adjustment and Root Architecture in Drought Conditions

MYB TFs are key regulators of osmotic homeostasis within and outside plant cells, mainly by facilitating the accumulation of small-molecule osmolytes, remodeling root system architecture, and modifying cell wall properties.^166^ OE lines of *IbMYB48* in *Ipomoea batatas* and *AhMYB44–11* in *Arachis hypogaea* significantly upregulated the expression of osmotic stress-responsive genes such as *LET14*, *P5CS*, and *DNH6*. This led to the accumulation of soluble sugars and proline, maintaining osmotic balance and membrane integrity, thereby enhancing drought tolerance.^167,168^ Plant roots are critical for sensing soil moisture changes and absorbing water and nutrients. Under water deficit conditions, changes in root architecture help plants access deeper soil water, supporting normal growth and development.^169^ MYBs actively regulate root development under drought stress. For example, *OsRRS1* in *Oryza sativa* binds to the promoter region of the auxin signaling repressor gene *IAA3*, suppressing its transcription and disrupting normal root development. Overexpression of *OsRRS1* resulted in shorter lateral roots, reduced lateral root density, and smaller root volume, impairing water absorption and utilization efficiency, thus negatively regulating drought tolerance.^170^ Similarly, *LbTRY* from *Limonium bicolor* was upregulated under drought stress, activating the transcription of *GL3/ZFP5* and competitively binding to its transcript. This interfered with the formation of the TTG1-GL3-GL1 complex, inhibiting trichome development while promoting root hair growth. This enhanced Na^+^ absorption but negatively impacted drought tolerance.^112^ Plants also modify cell wall composition and structure to maintain osmotic balance under drought stress. For instance, *MdMYB46* in *Malus domestica* directly binds to the promoters of lignin metabolism regulatory genes *MdMYB58* and *MdMYB63*, positively regulating lignin accumulation and improving osmotic stress tolerance.^139^ In *Populus tomentosa*, drought stress induced the expression of *PtoMYB142*, which enhanced the transcription of wax biosynthesis genes *CER4* and *KCS*. This led to increased wax accumulation on leaf surfaces, significantly easing water loss and refining drought tolerance.^171^

#### 3.2.3. Hormonal Crosstalk Modulated by MYBs During Drought Stress

MYBs participate in drought stress responses by modulating plant hormonal pathways. Particularly, ABA signaling induces ion efflux in guard cells, leading to stomatal closure, which minimizes water loss and thereby improves drought tolerance.^172^ For example, *SiMYB75* in *Sesamum indicum* significantly upregulated ABA biosynthesis genes *AtNCED3* and *AtABA3* under drought stress. Overexpression of *SiMYB75* in *Arabidopsis* increased endogenous ABA levels, activating drought-responsive genes *RD29B* and *RD22* and mediating stomatal closure via *AtOST1*, reducing water loss and enhancing drought tolerance.^173^ Similarly, *GhMYB44* in *Gossypium hirsutum* negatively regulates the expression of *HAB1*, *ABI1*, and *PP2CA*, promoting stomatal closure and improving drought tolerance.^174^ The overexpression of *ZmMYB3R* from maize in *Arabidopsis* promoted abscisic acid (ABA) biosynthesis, upregulated the expression of *RD29A/B, ABF3*, and *ABA1* genes, expedited stomatal closure, decreased transpiration rates, and consequently enhanced drought tolerance.^174^ Brassinosteroids (BR) also improved drought tolerance by contributing to osmotic regulation. *GmMYB14* in *Glycine max* bound to the AC *cis*-element in the *GmBEN1* promoter. Under drought stress, the transcription of *GmBEN1* was upregulated, accelerating BR catabolism and mitigating the inhibitory effect of BR signaling on drought responses, thereby enhancing drought tolerance.^175^

### 3.3. MYB Involvement in Temperature Stress Responses

#### 3.3.1. myb-Driven Mechanisms Under Cold Stress

**Figure 3.**
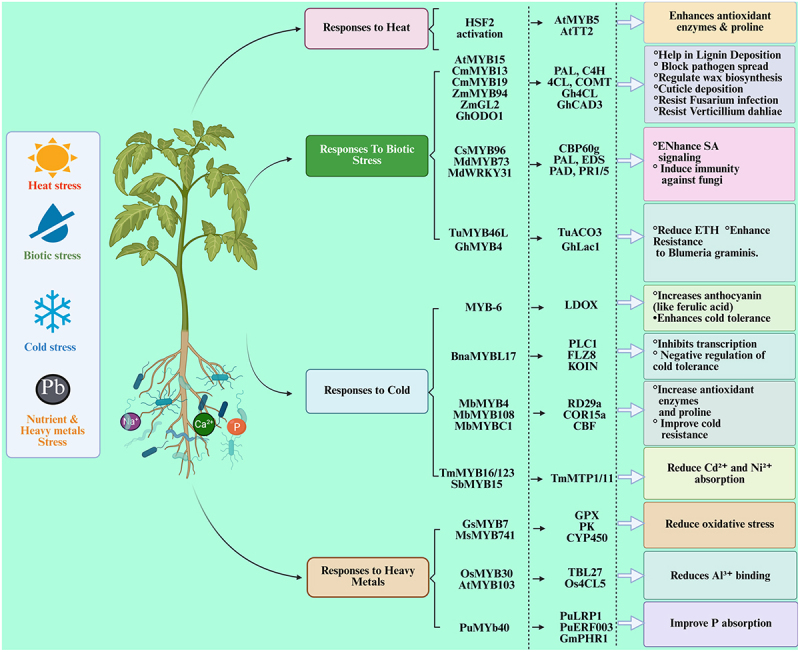
Schematic illustration of MYB transcription factors involved in plant responses to various stresses, including heat, cold, biotic, and heavy metal stress. Each stress type triggers specific MYB genes that regulate downstream signaling pathways and stress-responsive genes. These MYBs enhance tolerance by promoting antioxidant activity, secondary metabolite synthesis, lignin deposition, hormonal signaling, and ion homeostasis. Key gene interactions and stress outcomes are summarized. Colored arrows indicate functional pathways under each stress condition.

Low temperatures decrease the fluidity of plant cell membranes and impair enzyme activity, leading to the excessive accumulation of ROS, which prominently affects normal plant growth, development, and metabolic functions.^176,177^ MYBs participate in the response to cold stress by modulating both CBF-dependent and CBF-independent signaling pathways^178,179^ (Fig. 3). Research has shown that MYB regulates the expression levels of CBF transcription factors in the cold response pathway, which involves the inducer of CBF expression (ICE), CBF, and cold-regulated genes (COR), thereby affecting plant cold tolerance.^180^ Under cold stress, *MbMYB4, MbMYB108*, and *MbMYBC1* in *Malus baccata* activated the transcription of CBF cold stress response pathway-related genes, including *RD29a*, *COR15a*, and *CBF*. This improved antioxidant system and proline content, thereby improving plant cold resistance.^181–183^ However, some MYB transcription factors also regulate plant sensitivity to cold through CBF-independent pathways.^179,184^ For example, *BnaMYBL17* in *Brassica napus* binds to the promoter regions of *PLC1*, *FLZ8*, and *KOIN*, inhibiting their transcription and disrupting the balance between plant growth and cold response under cold conditions. Transgenic plants exhibit increased osmotic permeability, reduced soluble sugar and proline content, and enhanced sensitivity to cold, indicating that *BnaMYBL17* negatively regulates plant cold tolerance.^185^ Additionally, the crosstalk between MYB and anthocyanin biosynthesis also affects the tolerance to low temperatures.^186^ Compared to controls, cold treatment significantly increased the expression of *MYB-6* and *LDOX* in *Daucus carota*, along with a marked increase in anthocyanin content, including ferulic acid. This suggests that *MYB-6* may play a role in enhancing anthocyanin content under cold stress.^187^

#### 3.3.2. myb-Driven Mechanisms Under Heat Stress

Heat stress causes protein misfolding and leads to the unnecessary accumulation of ROS in plant cells.^188,189^ MYB transcription factors also participate in regulating a series of heat stress response genes to mitigate the damage caused by high temperatures^190,191^ (Fig. 3). Extensive research has shown that 12, 8, and 12 MYB family members in *Dendrobium catenatum*, *Liriodendron chinense*, and *Pearl millet*, respectively, play crucial roles in responding to high-temperature stress by reducing ROS levels and enhancing plant thermotolerance.^52,56,192^ Additionally, MYB transcriptionally regulates heat shock factors (HSFs), providing an effective pathway for plants to respond to heat stress. For example, *AtMYB5* and *AtTT2* in *A. thaliana* bind to the SIIE motif in the HSF2 promoter, upregulating HSF2 expression, enhancing antioxidant enzyme activity, and improving tolerance to high-temperature stress.^193^

### 3.4. MYB Functions in Nutrient and Heavy Metal Stress

#### 3.4.1. MYB Regulation of Nutrient Uptake and Allocation

MYB TFs are critical regulators of plant root nutrient uptake capacity, particularly under conditions of nutrient deficiency.^194^ Phosphorus (P) is a vital element for plant growth and development.^195^ Phosphorus deficiency can hinder crop growth and decrease yield.^196^ MYBs are involved in mediating plant responses to low phosphorus stress^197,198^ (Fig. 3). For instance, *PuMYB40* in *Populus ussuriensis* interacts with *PuWRKY75* to enhance the transcription of low phosphorus-responsive genes *PuLRP1* and *PuERF003*, thereby facilitating the development of adventitious roots.^199^ Similarly, MYB-like transcription factors, known as phosphate starvation response (PHR) proteins, are key components of phosphorus signaling and play a role in processes such as metal element absorption.^200^ Overexpression of *GmPHR1* from soybean significantly increased the number of lateral roots and root nodules, enhanced the symbiotic nitrogen fixation ability of rhizobia, and promotes the absorption of nitrogen and phosphorus, thereby increasing seed yield.^201^ Boron (B) is an essential micronutrient for plant growth, and its deficiency can result in poor plant development.^202^ MYBs also contribute to maintaining normal growth and development under boron-deficient conditions.^203^ In *Pyrus betulaefolia*, the expression of nine MYBs was significantly upregulated under short-term boron deficiency. These genes enhanced the root’s ability to absorb boron and integrated ABA and JA signaling pathways to regulate the plant’s response to boron stress.^204^

#### 3.4.2. MYB Transcription Factors in Heavy Metal Homeostasis

Heavy metals can severely affect plant growth and development by causing an excessive buildup of ROS, lowering photosynthetic efficiency, and damaging cell membranes.^188,205,206^ Furthermore, heavy metals may infiltrate the human body via the food chain, causing long-term and often irreversible damage.^207^ Research has shown that MYBs can enhance plant tolerance to heavy metals by regulating the absorption of these elements or mitigating the oxidative damage they cause^208,209^ (Fig. 3). Under aluminum (Al) stress, *OsMYB30* functions as a negative regulator of Al tolerance in rice. By binding to the promoter of Os4CL5, it reduces the accumulation of *p*-coumaric acid (PA) in the cell wall, thereby increasing the binding of Al^3 +^ to the cell wall and significantly lowering Al tolerance. However, the O*sMYB30-Os4CL5* pathway is negatively regulated by the upstream factor *OsART1*, which alleviates the aluminum-sensitive phenotype caused by *OsMYB30*.^210^ In contrast, *AtMYB103* from *Arabidopsis* functions as a positive regulator of aluminum tolerance by upregulating the expression of *TBL27*, a gene involved in cell wall xyloglucan O-acetylation. This modification decreases Al^3 +^ binding to the cell wall, thereby substantially improving aluminum tolerance in transgenic plants.^211^ MYBs also play a role in regulating the antioxidant capacity of plants under heavy metal stress.^75^ For instance, overexpression of *GsMYB7* from *Glycine soja* significantly increases the total root surface area of transgenic plants, reduces the accumulation of Al^3 +^ in root tips, and upregulates the expression of genes encoding glutathione peroxidase (GPX), protein kinases (PK), and cytochrome P450 (CYP450), thus mitigating oxidative damage and enhancing aluminum tolerance.^212^ Similarly, overexpression of *TmMYB16/123* from *Taxus media* and *SbMYB15* from *Salicornia brachiata* in transgenic plants exposed to Cd^2 +^ and Ni^2 +^ effectively inhibits the transcription of heavy metal ion transporters *TmMTP1/11*, significantly limits the absorption of Cd^2 +^ and Ni^2 +^, reduces ROS accumulation, and increases the activity of antioxidant enzymes such as SOD, POD, and CAT, thereby enhancing plant tolerance to heavy metals.^213,214^ Additionally, *MsMYB741* from *Medicago sativa* influences the flavonoid biosynthesis pathway in transgenic plants, resulting in the accumulation of antioxidant flavonoids that effectively scavenge intracellular ROS and positively regulated aluminum tolerance.^215^

### 3.5. MYB Regulatory Role in Plant Defense Against Biotic Stress

Apart from their critical regulatory role in plant responses to abiotic stress, the MYB family is also extensively involved in regulating plant immune functions under various biotic stresses, enhancing plant defense mechanisms against pathogenic microorganisms (fungi, bacteria, viruses) and herbivorous insects.^216^ Plant MYBs primarily confer tolerance to biotic stress by modulating leaf cuticle thickness and participating in the regulation of multiple plant hormone signaling pathways (Fig. 3).

#### 3.5.1. Strengthening Cell Wall Barriers: Lignin and Cuticle Deposition

The plant cell wall acts as the primary natural barrier against pathogen invasion.^217^ Modulating the components of the cell wall can strengthen the plant’s defense response to pathogenic attacks.^218,219^ In many plants, MYBs have been found to play key role in the biosynthesis of lignin, cuticular wax, and other substances, as well as the response to pathogen infection.^75,220^ Signification has been proven to positively correlate with plant disease resistance by restricting the movement of pathogens, thereby preventing their spread.^221^ In *A. thaliana*, *AtMYB15* activates lignin biosynthesis genes (*PAL, C4H, 4CL*, and *COMT*), leading to increased lignin content, which restricts P*seudomonas syringae* pv. tomato DC3000 to the infection site and triggers the plant’s immune response.^222^ In *Chrysanthemum morifolium*, *CmMYB13* and *CmMYB19* are induced by aphid attack and bind to AC elements in the promoters of lignin biosynthesis genes (*PAL*, *C4H*, *4CL*, *HCT*, and *CCR*), enhancing lignin accumulation to limit aphid growth and reproduction on *chrysanthemum*.^223,224^ Similarly, the *R2R3*-type MYB transcription factor *GhODO1*, identified in cotton, is induced by *Verticillium dahliae* and directly activates the transcription of *Gh4CL* and *GhCAD3*, enhancing cotton’s resistance to *Verticillium dahliae*.^225^ In maize, *ZmMYB94* and *ZmGL2* regulate the elongation of fatty acid chains, which are precursors of wax on the silk surface, and contribute to cuticle deposition, thereby enhancing resistance to *Fusarium verticillioides*.^226^

#### 3.5.2. myb-Mediated Activation of Biotic Stress Signaling Pathways

Polysaccharide signal transduction is also an essential mechanism for plants to manage biotic stress.^227,228^ In cotton, *GhMYB4* binds to the *GhLac1* promoter, inhibits its expression and significantly increases the content of oligogalacturonides in the cell wall. Membrane surface receptors recognize polysaccharide signals and further stimulate the immune system. Thus, *GhMYB4* enhances resistance to *Verticillium dahliae* through polysaccharide signaling.^229^ Crosstalk between single or multiple plant hormone signaling pathways is also vital in plant responses to biotic stress.^230,231^ In *Triticum urartu*, *TuMYB46L* functions as a transcriptional repressor of *TuACO3*, reducing the increase in ethylene (ETH) synthesis triggered by *Blumeria graminis* infection. Silencing *TuMYB46L* significantly enhances wheat’s tolerance to *Blumeria graminis*. The *TuMYB46L-TuACO3* module promotes wheat’s defense against *Blumeria graminis* by regulating ETH biosynthesis.^232^ In blood orange, *CsMYB96*, and in apple, *MdMYB73* directly activate the expression of salicylic acid (SA) biosynthesis genes (*CBP60g, PAL, EDS*, and *PAD*) and SA signaling genes (*PR1/5*), boosting SA biosynthesis, reducing ROS levels at fungal infection sites, and triggering programmed cell death. Additionally, *MdWRKY31* interacts with *MdMYB73* to promote the stimulation of transcription of target genes, improving transgenic plant resistance to *Penicillium italicum*, *Botrytis cinerea*, and *Botryosphaeria dothidea*.^233,234^

## 4. Regulatory Roles of MYB TFs in Secondary Metabolism

The co-evolution of plants with their environments has driven the diversification and production of a wide array of secondary metabolites, which are fundamental for plant adaptation and survival.^235^ These metabolites encompass monolignols, phenolic acids, stilbenes, and various flavonoids-including anthocyanins, proanthocyanidins, flavanones, flavonols, and isoflavonoids, that play indispensable roles in plant defense, signaling, and environmental adaptation.^33,236–239^ Beyond their defensive functions against pathogenic microbes, these secondary metabolites also contribute broadly to the regulation of plant metabolic pathways.^240^ Key components that reinforce mechanical strength, such as cell wall constituents, signaling molecules, pigments, UV-absorbing compounds, and phytoalexins are vital in modulating plant responses to diverse biotic and abiotic stresses.^241^ Among the transcriptional regulators, MYB (Myeloblastosis) transcription factors represent one of the largest and most significant families in plants, critically governing gene expression.^242^ Their role in secondary metabolite biosynthesis has been extensively highlighted, underscoring their functional importance in plant metabolic networks.^243^ Furthermore, the evolutionary conservation of MYB TF structures and functions across plants, yeast, and mammals emphasizes their essential regulatory roles across kingdoms.^244^ Current studies on MYB TF-mediated regulation of secondary metabolism have largely focused on economically important crops such as rice, apple, and soybean.^245^

**Figure 4.**
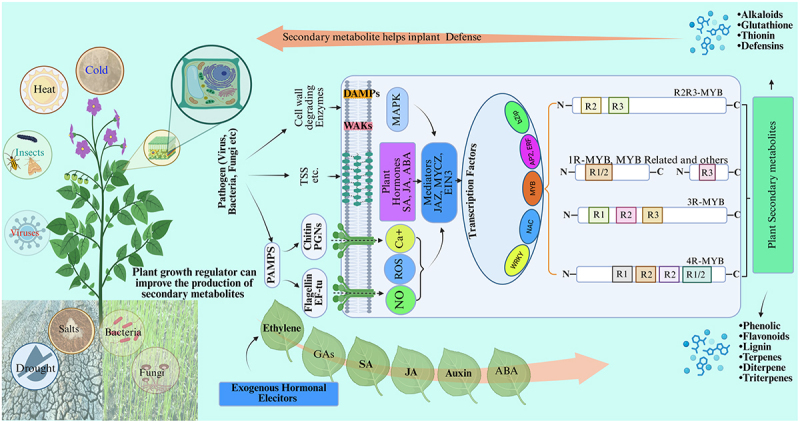
Schematic illustration of plant response mechanisms to biotic and abiotic stresses, highlighting the role of MYB transcription factors in regulating secondary metabolite biosynthesis. Environmental stimuli such as drought, salinity, cold, pathogens, and herbivores activate complex signaling pathways involving PAMPs, DAMPs, MAPKs, ROS, NO, and Ca^2 +^. These pathways are modulated by phytohormones (SA, JA, ABA, ET, GAs, auxin) and exogenous elicitors, leading to the activation of key transcription factors including MYB, WRKY, bHLH, NAC, and AP2/ERF. MYBs, classified into 1R-, 2R-, 3R-, and 4R-MYBs based on their DNA-binding domain repeats, play a central role in modulating the expression of genes involved in the biosynthesis and accumulation of secondary metabolites such as flavonoids, alkaloids, terpenes, and lignin, thereby enhancing plant stress tolerance and defense.

Several MYB TFs have been identified as key modulators of specific secondary metabolic pathways. For example, *VcMYB4a* in *Vaccinium corymbosum*,^246^ *PvMYB4a in Panicum virgatum*,^247^ and *ZmMYB31* and *ZmMYB42* in *Zea mays*,^248^ are known as repressors of lignin biosynthesis. In *Malus domestica*, MYB TFs such as *MdMYB1, MdMYB3*, and *MdMYBA* regulate the biosynthesis and accumulation of red anthocyanin pigments in fruit peel.^245^ In *Arabidopsis thaliana*, *AtMYB111, AtMYB12*, and *AtMYB11* individually activate genes encoding key flavonol biosynthetic enzymes-including flavonol synthase (FLS), flavanone 3-hydroxylase (F3H), chalcone isomerase (CHI), and chalcone synthase (CHS), which enhance the plant’s resilience to various stresses.^249^ Structurally, MYB TFs typically contain one to four imperfect repeat motifs within their DNA-binding domains, categorizing them into four subgroups: 1 R-MYB, 2 R-MYB, 3 R-MYB, and 4 R-MYB.^28,248^ These factors regulate a broad spectrum of plant functions, including cellular morphogenesis, metabolic processes, and environmental stress adaptation.^250^ In *Dendrobium candidum*, 117 2 R-MYB genes have been identified, with nine linked specifically to heat stress response.^245^ Under saline stress, multiple MYB TFs such as *DoMYB28, DoMYB29, DoMYB54, DoMYB75, DoMYB78, DoMYB81*, and *DoMYB111* are upregulated, indicating their involvement in stress tolerance.^245^ In *Betula platyphylla, BplMYB46* interacts with GT-box, E-box, and TC-box motifs in promoters of stress-responsive genes including superoxide dismutase (SOD), peroxidase (POD), and phenylalanine ammonia-lyase (PAL), as demonstrated by co-expression reporter assays and chromatin immunoprecipitation in *Nicotiana tabacum*.^251^ These genes are critical for secondary cell wall biosynthesis and contribute to stress resilience.^243^ In addition, MYB transcription factors are key contributors to flavonoid biosynthesis, influencing plant development by interacting with hormonal pathways, responding to environmental cues, engaging with microRNAs, and forming complexes with other transcription factors. Through these interactions, they integrate into intricate regulatory networks that coordinate growth and stress responses in plants^22,252^ (Fig. 4).

## 5. Research Gaps, Technical Challenges, and Future Directions

Despite significant advances in understanding the roles of MYB transcription factors (TFs) in plant stress responses, several critical gaps continue to limit their full exploitation in crop improvement.^253^ A major obstacle is the functional redundancy and genetic compensation among MYB family members, which share highly conserved DNA-binding domains and overlapping target genes. This redundancy often masks phenotypic effects in knockout or silencing studies, complicating the elucidation of specific MYB functions.^254,255^ Additionally, MYB transcription factors display functional diversity across species, tissues, and developmental stages, but the molecular mechanisms underlying this context-dependent regulation remain unclear.^75,254^ The comprehensive interaction networks and post-translational modifications regulating MYB activity under stress are still underexplored. Moreover, integration of MYB-mediated transcriptional regulation with hormone signaling, epigenetic modifications, and other transcription factor families during multi-stress responses requires further investigation.^68,255^ Emerging evidence also suggests that MYBs may participate in non-canonical regulatory pathways such as RNA metabolism and epigenetic regulation, but these roles remain largely uncharacterized.^28,68,255^

Technical challenges further restrict progress in MYB research. Traditional gene knockout or RNA interference approaches often fail to reveal clear MYB functions due to redundancy.^75,255^ However, advanced genome editing tools like CRISPR/Cas multiplexing hold promise for overcoming this challenge. Detailed expression profiling at single-cell or tissue-specific levels under dynamic stress conditions is limited, restricting precise functional annotation.^256^ Functional characterization is heavily biased toward model plants such as *Arabidopsis* and rice, with limited validation in economically important crops, especially under field conditions. Additionally, the complexity of multi-stress and multi-signal integration complicates research, as most studies focus on single stress conditions, whereas natural environments impose combined stresses.^257^

In addition to the well-established roles of MYBs, growing evidence suggests many novel and uncharacterized functions remain to be discovered.^258^ Certain MYB members may participate in non-canonical regulatory pathways, including RNA metabolism, epigenetic modifications, or cross-talk with hormone signaling beyond the classical ABA, JA, and SOS pathways.^68^ Exploring these potential roles could uncover new layers of gene regulation and stress adaptation in plants, opening exciting avenues for future research.^259^ Some MYB proteins, such as *AtMYB44, AtMYB15*, and *OsMYB30*, have emerged as central regulatory hubs involved in multiple physiological and stress-related processes, including abiotic and biotic stress responses, growth, and development.^245^ These multifunctional MYBs represent promising candidates for breeding programs aimed at enhancing crop resilience and productivity. However, their pleiotropic effects, such as developmental delays or dwarfism, necessitate careful evaluation and precise genetic manipulation to avoid undesirable phenotypes.^75,254,260^

To address current gaps and harness the full potential of MYBs, future research should emphasize comprehensive functional genomics approaches using CRISPR/Cas-mediated multiplex gene editing combined with transcriptomics and proteomics to dissect gene family redundancy and reveal unique versus overlapping functions.^254^ Elucidation of MYB regulatory networks through chromatin immunoprecipitation sequencing (ChIP-seq), yeast two-hybrid systems, bimolecular fluorescence complementation (BiFC), and protein modification analyses will be crucial to mapping interactomes and regulatory circuits under various stresses.^261^ High-resolution expression profiling using single-cell RNA sequencing and tissue-specific analyses under dynamic stress conditions will refine functional annotation. Integrating multi-omics data with computational modeling will help unravel MYB-mediated cross-talk among hormonal, metabolic, and stress signaling pathways.^262^ Expanding research to diverse crop species and validating MYB functions in realistic agricultural environments is essential for translational applications.^263^ Investigating emerging non-canonical functions of MYBs in RNA metabolism and epigenetic regulation, along with developing tissue-specific and inducible expression systems, will enable precise genetic engineering while minimizing pleiotropic effects.^260^ Ultimately, leveraging these insights through breeding and biotechnological approaches will facilitate engineering crops with enhanced tolerance to combined abiotic and biotic stresses, improving yield and resilience to climate change. Addressing these priorities will be pivotal for advancing sustainable agriculture and food security.^264^

## 6. Conclusion

MYB transcription factors constitute a pivotal family of regulatory proteins that coordinate plant responses to diverse environmental challenges, including salinity, drought, temperature extremes, nutrient deficiencies, heavy metal toxicity, pathogen attacks and pest infestations. Through specific binding to promoter regions, MYBs regulate complex gene networks involved in maintaining ionic and osmotic balance, enhancing antioxidant defenses, remodeling cell walls, and modulating hormone signaling pathways such as abscisic acid (ABA). These multifaceted roles enable plants to adapt effectively to adverse environmental conditions by preserving cellular integrity, optimizing water use, and activating defense mechanisms. Research has demonstrated that MYBs can act as both positive and negative regulators depending on the stress context, underscoring their intricate and dynamic functions. Additionally, MYBs are integral to nutrient uptake, heavy metal detoxification, and immune responses through regulation of lignification, cuticle formation, and hormone-mediated signaling. Despite substantial progress, many MYB family members remain functionally uncharacterized, and the complexity of their regulatory networks, including interactions with other transcription factors and involvement in epigenetic and non-canonical pathways, requires further investigation. Challenges such as gene redundancy and context-dependent activity highlight the need for precise functional dissection. Ultimately, leveraging the regulatory potential of MYB transcription factors through advanced genomics and biotechnological approaches offers promising avenues for engineering stress-resilient crops, which is essential for ensuring sustainable agriculture and food security amid increasing environmental challenges.
